# Biological nitrogen fixation in cereal crops: Progress, strategies, and perspectives

**DOI:** 10.1016/j.xplc.2022.100499

**Published:** 2022-11-28

**Authors:** Kaiyan Guo, Jun Yang, Nan Yu, Li Luo, Ertao Wang

**Affiliations:** 1 Shanghai Key Laboratory of Plant Molecular Sciences, College of Life Sciences, Shanghai Normal University, Shanghai 200234, China; 2 National Key Laboratory of Plant Molecular Genetics, CAS Center for Excellence in Molecular Plant Sciences, Institute of Plant Physiology and Ecology, Chinese Academy of Sciences, Shanghai 200032, China; 3 School of Life Sciences, Shanghai Key Laboratory of Bioenergy Crops, Shanghai University, Shanghai 200444, China

**Keywords:** biological nitrogen fixation, cereal plants, self-fertilizing crops, microbiome

## Abstract

Nitrogen is abundant in the atmosphere but is generally the most limiting nutrient for plants. The inability of many crop plants, such as cereals, to directly utilize freely available atmospheric nitrogen gas means that their growth and production often rely heavily on the application of chemical fertilizers, which leads to greenhouse gas emissions and the eutrophication of water. By contrast, legumes gain access to nitrogen through symbiotic association with rhizobia. These bacteria convert nitrogen gas into biologically available ammonia in nodules through a process termed symbiotic biological nitrogen fixation, which plays a decisive role in ecosystem functioning. Engineering cereal crops that can fix nitrogen like legumes or associate with nitrogen-fixing microbiomes could help to avoid the problems caused by the overuse of synthetic nitrogen fertilizer. With the development of synthetic biology, various efforts have been undertaken with the aim of creating so-called “N-self-fertilizing” crops capable of performing autonomous nitrogen fixation to avoid the need for chemical fertilizers. In this review, we briefly summarize the history and current status of engineering N-self-fertilizing crops. We also propose several potential biotechnological approaches for incorporating biological nitrogen fixation capacity into non-legume plants.

## Teaser

Legumes can gain access to nitrogen through symbiotic association with rhizobia, whereas the growth and production of cereal crops often rely heavily on the application of chemical fertilizers. Engineering cereal crops that can fix nitrogen autonomously or that associate with nitrogen-fixing bacteria through a synthetic biology approach would reduce the use of synthetic nitrogen fertilizers. This review summarizes the history and current status of engineering biological nitrogen fixation in cereal plants.

## Introduction

**Figure 1:**
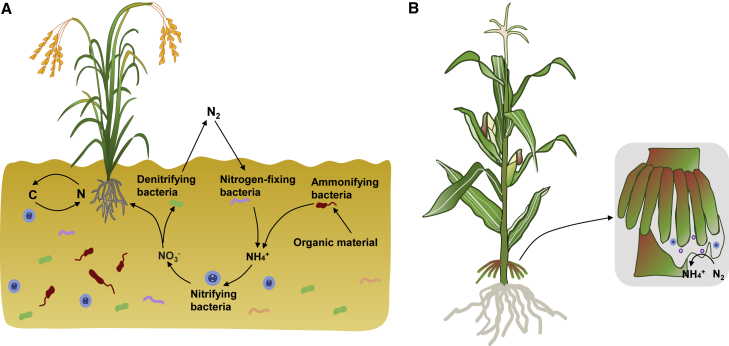
Nitrogen-cycling and biological nitrogen fixation from microbiomes associated with cereal crops. **(A)** Nitrogen-cycling process in the rhizosphere of cereal crops. The nitrogen cycle is a biogeochemical process that includes the conversion of atmospheric nitrogen gas into organic nitrogen, which is available to living organisms. The nitrogen is subsequently returned to the atmosphere. The complete nitrogen cycle can be divided into four steps: nitrogen fixation, assimilation, nitrification, and denitrification. During nitrogen fixation, inert atmospheric nitrogen is converted into a usable form of nitrogen (NH_4_^+^) by symbiotic bacteria, e.g., rhizobia. Organic remains from plants and animals in the soil gradually decay and ammonify with the help of ammonifying bacteria. During nitrification, nitrite is oxidized to form inorganic nitrogen (NO_3_^−^) via the action of nitrifying bacteria. The NO_3_^−^ can be absorbed and utilized by plants. Some nitrates are not absorbed by plants and can be reduced to nitrite by microorganisms such as denitrifying bacteria and further reduced to molecular nitrogen, which is returned to the atmosphere. **(B)** The aerobic roots of Sierra Mixe maize secrete mucilage for BNF. The mucilage is rich in carbohydrates and provides a low-oxygen microenvironment for the nitrogenase activity of diazotrophic microbiota.

Legume crops such as soybean (*Glycine max*) and pea (*Pisum sativum*) represent major sources of protein for livestock and humans, whereas cereal crops such as maize (*Zea mays*), wheat (*Triticum aestivum*), and rice (*Oryza sativa*) are major sources of carbohydrates. High-yield cereal crops generally require the application of chemical nitrogen (N) fertilizers, whereas legumes can form symbiotic root nodules with rhizobia to secure the N needed for growth by biological N fixation (BNF). The increasing demand for food driven by global population expansion necessitates major increases in agricultural production, which currently relies heavily on high-yielding crop varieties and chemical N fertilizers. The Food and Agriculture Organization estimates that maize, wheat, and rice together account for approximately 80% of all grain production worldwide. Chemical N fertilizers must be supplied to these crops throughout their growth periods to achieve adequate yields. However, the N use efficiency of cereals has dropped to approximately 34% (Food and Agriculture Organization, 2022), and the remaining ∼56% of N fertilizer is lost to land and water or reduced to nitrous oxide by denitrification (Skiba, 2008). This leads to serious environmental problems such as soil compaction, acidification, the release of heavy metal ions, microflora imbalances, and increasing greenhouse gas emissions (Ahmed et al., 2017), which threaten the sustainable development of agriculture and food security. Thus, improving the capacity for BNF in cereal crops would help to solve these serious problems. BNF is the process by which nitrogenase in prokaryotes converts dinitrogen gas from the atmosphere into ammonia under anaerobic/microaerobic conditions at normal temperature and atmospheric pressure (Wagner, 2011; Figure 1A).

Before the invention of Haber’s synthetic ammonia technology, which requires high temperature and high pressure for artificial N fixation, combined N in nature was mainly obtained from BNF by prokaryotes (including bacteria and archaea), lightning, or volcanic eruptions (Soumare et al., 2020). There are three forms of BNF: free living, associative, and symbiotic (Olivares et al., 2013; Pankievicz et al., 2019; Soumare et al., 2020). In free-living N fixation, diazotrophs, including microorganisms such as *Nostoc flagelliforme*, *Azotobacter vinelandii*, and *Paenibacillus sabinae*, fix N for their own use under microaerobic conditions (Pankievicz et al., 2019; Soumare et al., 2020). In associative N fixation, diazotrophs living on the surfaces or in the interstitial spaces of their plant host use photosynthetic products from the plant as carbon sources to fix N for their own use, providing the excess to the host; these microorganisms include *Azospirillum brasilense* and *Pseudomonas stutzeri* (Pankievicz et al., 2019; Soumare et al., 2020). The annual worldwide N fixation by associative N-fixing bacteria is approximately 50–70 teragrams (Tg). In symbiotic N fixation, diazotrophs in the cells of plant organs such as root nodules use photosynthetic products from the host as energy sources and fix N to support host growth and development. Examples include the rhizobium/legume, *Frankia*/alder, and cyanobacteria/cycad systems (Pankievicz et al., 2019; Soumare et al., 2020). The annual worldwide N fixation by the rhizobia–legume symbiosis is approximately 21.5 Tg (Bueno Batista and Dixon, 2019; Pankievicz et al., 2019).

Efforts have been undertaken to engineer non-legume plants with the capacity for N fixation (to make so-called “N-self-fertilizing” crops) using synthetic biology approaches (reviewed by Santi et al., 2013; Rogers and Oldroyd, 2014; Oldroyd and Dixon, 2014; Mus et al., 2016; Mathesius et al., 2021; Sheoran et al., 2021; Shamseldin, 2022). In this review, we briefly summarize progress toward engineering BNF capacity in cereal crops and propose several biotechnological approaches for the incorporation of BNF systems into cereal plants. These new synthetic biology approaches could reduce our dependency on chemical N fertilizers and help to create N-self-fertilizing plants.

### BNF by microbiomes associated with cereal crops

N-fixing prokaryotes fix atmospheric dinitrogen gas into ammonia using a relatively conserved nitrogenase complex consisting of a dinitrogen synthase, a dinitrogen synthase reductase, and metal co-factors (Bulen and LeComte, 1966). Dinitrogen synthases can be divided into three groups based on the binding of different metal ions by active site co-factors: molybdenum (Mo) nitrogenase (the major nitrogenase component in N-fixing bacteria and archaea), vanadium nitrogenase, and iron (Fe) nitrogenase (Choudhary and Varma, 2017). The dinitrogen synthase Mo nitrogenase is formed from the *nifD* and *nifK* gene products, and dinitrogen synthase reductase is a homodimer of the *nifH* gene product (Shah and Brill, 1977). In addition to *nifH*, *nifD*, and *nifK*, genes involved in N fixation in many N-fixing bacteria also include *nifE*, *nifN*, *nifX*, *nifQ*, *nifW*, *nifV*, *nifA*, *nifB*, *nifZ*, and *nifS*, which are involved in biosynthesis and assembly of FeMo co-factors, regulation of *nif* gene expression, and maturation of the electron transfer chain (Lee, et al., 2000; Masepohl et al., 2002). The *fixABCX* genes found in rhizobia encode a membrane complex involved in electron transfer to nitrogenase (Kallas et al., 1985; Earl et al., 1987; Edgren and Nordlund, 2004); these genes are absent in free-living and endophytic bacteria that fix N.

Free-living N-fixing bacteria in the rhizosphere of cereal crops, as well as endophytic N-fixing bacteria, use a carbon source and energy provided by the environment (root-excreted carbohydrates, decomposing soil organic matter, or plant photosynthetic products) to fix N. Bacteria release fixed N to provide partial combined N for plant growth and development (James, 2000; White et al., 2012). These bacteria, including *Azotobacter*, *Azospirillum*, *Bacillus*, *Clostridium*, and *Rhodospirillum* species (Sawada et al., 2003), contribute combined N to the biosphere, providing 30%–50% of total fixed N in some farmland ecosystems (Rosenblueth et al., 2018). Various bacteria of the *Azospirillum*, *Bacillus*, *Paenibacillus*, *Burkholderia*, and *Gluconobacter* genera can be used as endophytes for associative N fixation in crops such as rice, banana, maize, and sugarcane (*Saccharum officinarum*) (Nadarajah and Abdul Rahman, 2021). The endophytic N-fixing bacteria *Azorhizobium caulinodans* and *G. diazotrophicus* promote the growth of maize, wheat, and sugarcane. *Setaria viridis* and *Setaria italica* (millet) can obtain substantial amounts of N from associative N fixation with *A. brasilense* (Okon et al., 1983; Pankievicz et al., 2015). The annual amounts of N fixed by bacteria associated with rice and sugarcane are approximately 5 and 0.5 Tg, respectively (Herridge et al., 2008). Approximately 20%–25% of the N requirements of both rice and maize can be met through associative N fixation (Montañez et al., 2012).

Some rhizobia fix N as free-living bacteria in the rhizosphere or as endophytes in cereal crops. Most rhizobia possess *nodABC* genes encoding enzymes required for the synthesis of lipo-chitooligosaccharides (also called Nod factors [NFs]) that induce symbiotic responses in host legumes. However, some rhizobial strains use an NF-independent infection process (Giraud et al., 2007; Bonaldi et al., 2010; Chaintreuil et al., 2013) of “crack entry” to infect the stem xylem parenchyma of plants such as sugarcane for N fixation (Thaweenut et al., 2010). These bacteria fix N under hypoxic or microaerobic conditions. Examples of nodule-inducing rhizobia are *Bradyrhizobium* (which forms nodules on both legume *Aeschynomene* and non-legume *Parasponia* species), *A. caulinodans* (which forms nodules on *Sesbania* species), and *Burkholderia* (which forms nodules on *Mimosa* species) (Dreyfus et al., 1983; Mohapatra et al., 1983; Gebhardt et al., 1984; Alazard, 1990; Chen et al., 2003; Elliott et al., 2007).

#### Rice

Rice is an important grain that is widely distributed in tropical and temperate regions of Asia. The rice root–soil interface is used as a N-fixing site in flooded soils. Bacteria that maintain this N-fixing activity under dark, flooded conditions, such as *Azotobacter*, *Clostridium*, *Herbaspirillum*, and *Azospirillum*, are considered to be heterotrophic N-fixing bacteria (Yoshida and Ancajas, 1973; Kimura et al., 1979; Ishii et al., 2011). During long-term repeated pot experiments at the International Rice Research Institute, N fixation was observed by both photosynthetic cyanobacteria and heterotrophic diazotrophs, which use the carbon source secreted by the rice root system into the rhizosphere (App, et al., 1980; Yoneyama et al., 2017). A positive N balance was observed, indicating significant input of atmospheric N into rice fields (Ladha et al., 2016). Using crude DNA fragments from roots, *nifD* genes homologous to those of γ-proteobacteria (*A. vinelandii*) and α-proteobacteria (*B. japonicum*) were detected in rice fields (Ueda et al., 1995a; 1995b). Chemical N fertilizer, plant genotype, and other environmental conditions affect the diversity of the *nifH* gene pool in rice roots (Tan et al., 2003). Application of the trace element Mo increased the number of *nifH* gene copies, the relative abundance of cyanobacteria (such as *Leptolyngbya* and *Microcoleus* species), and BNF in Mo-deficient paddy fields (Ma et al., 2019). Notably, methanotrophs (such as *Methylosinus* and *Methylocystis*) function as active endophytic dinitrogen-gas-fixing bacteria in the roots of paddy-grown rice in methane-rich environments (Yoneyama et al., 2019) by colonizing the intercellular spaces around the root steles (Yoneyama et al., 2019); these bacteria were also detected in the microbiome associated with rice roots (Edwards et al., 2015; Ding et al., 2019; Mahmud et al., 2020).

#### Maize

Maize is an important C4 plant with high photosynthetic efficiency that is native to Central and South America. This crop is widely planted in tropical and temperate regions worldwide. Maize is a high-yielding food crop and an important source of feed for animal husbandry and aquaculture. Inoculation of maize with N-fixing bacteria such as *Pseudomonas* species and *Bacillus megaterium* produced the same dry weight and chlorophyll content as the addition of 33% N fertilizer (Kifle and Laing, 2016). Several diazotrophic bacteria function in N fixation in maize by establishing rhizospheric or endophytic associations, such as *Azospirillum*, *Klebsiella*, *Pantoea*, *Herbaspirillum*, *Rhizobium etli,* and *Burkholderia* (Dong et al., 2001; Gutiérrez-Zamora and Martínez-Romero, 2001; Caballero-Mellado et al., 2004; Perin et al., 2006).

Sierra Mixe, a maize variety grown in Mexico, produces mucilage around its aerobic roots, which contain many bacterial strains with active *nif* genes (Van Deynze et al., 2018). The role of this mucilage is to maintain oxygen levels below 5% at a depth of 8 mm, which is required for proper nitrogenase activity (Bennett et al., 2020). Using such mucilage, this maize variety meets 29%–82% of its N requirements through the activity of associative N-fixing bacteria. Small amounts of mucilage were recently detected in wheat, barley, and sorghum, suggesting that mucilage may be a common feature of cereal roots (Bennett et al., 2020; Figure 1B). A new maize hybrid with aerial roots and mucilage might be engineered to fix N in the near future.

γ-Proteobacteria, a highly conserved class of core members of the bacterial microbiota with N-fixation capacity, inhabit the xylem sap of maize plants, as recently reported by Zhang et al. (2022). The authors established a synthetic community consisting of two core diazotrophs and two helpers, which contributed 11.8% of the total N accumulated in maize stems via BNF. These core taxa in xylem sap represent an untapped resource that could be exploited to increase crop productivity.

#### Wheat

Wheat is the most extensively grown cereal crop in the world. The wheat caryopsis can be used to make bread, steamed bread, biscuits, noodles, and other staple foods. Inoculation with the N-fixing bacterium *Klebsiella pneumoniae* 342 alleviated symptoms of N deficiency in wheat and increased total N and N concentrations in wheat plants (Iniguez et al., 2004). ^15^N isotope tracking experiments confirmed that *Klebsiella pneumoniae 342* can fix N in wheat plants. Two bacterial strains isolated from the rhizosphere, *Bacillus subtilis* HG-15 and *Enterobacter cloacae* HG-1, enhanced the salt tolerance of inoculated wheat plants and promoted plant growth via BNF (Ji et al., 2020, 2022). Co-inoculation with a diazotrophic bacterium (*Paenibacillus beijingensis* BJ-18) and a phosphate-solubilizing bacterium (*Paenibacillus* sp. B1) significantly increased plant biomass, plant N content (∼30%), soil total N (12%), and nitrogenase activity (69%), confirming that N-fixing *P. beijingensis* is associated with wheat (Li et al., 2020). However, the N-fixation potential of the microbiome from wheat plants and the rhizosphere has not been assessed.

#### Sugarcane, sorghum, and millets

Sugarcane, a temperate and tropical crop, provides raw material for the manufacturing of sucrose and the extraction of ethanol as an energy source. *Beijerinckia* species were first isolated from the sugarcane rhizosphere at EMBRAPA (Brazilian Agricultural Research Corporation) Agrobiology in Brazil (Dobereiner, 1961). *nifH* gene expression was detected in cut stems of sugarcane plants that were grown in Japanese soil for 50 and 100 days by RT–PCR and *nifH* DNA sequencing (Thaweenut et al., 2010). These *nifH* sequences were similar to those of the N-fixing bacteria *Bradyrhizobium* and *A. caulinodans*, suggesting that free-living rhizobia may be a key factor in endophytic N fixation. In recent years, a variety of bacteria such as *Raoultella* sp. L03, *Microbacterium* sp. 16SH, *Enterobacter* spp. NN145S/NN143E, *Pantoea agglomerans* 33.1, *Stenotrophomonas pavanii* ICB89, *Bacillus megaterium* CY5, and *B. mycoides* CA1 have been identified from sugarcane plants and rhizospheres and shown to provide fixed N for plant growth and development (Ramos et al., 2011; Lin et al., 2012a; 2012b; Quecine et al., 2012; Luo et al., 2016; Singh et al., 2020).

Sorghum, another important C4 plant, is the fifth most widely cultivated cereal crop in the world (Taylor et al., 2006). N-fixation activity was first detected from washed root segments of sorghum by Pedersen et al. (1978). Several diazotrophic bacteria like *Paenibacillus*, *Azohydromonas*, *Ideonella*, *Rhizobium*, and *Bradyrhizobium* were identified in the rhizosphere soils of field-grown sorghum (Coelho et al., 2008). Bacterial communities from sorghum were characterized with respect to their metagenomes and proteomes. The major functional N-fixing bacteria in sorghum roots were found to be unique non-nodulating or photosynthetic bradyrhizobia (*Bradyrhizobium sp.* S23321; *B. oligotrophicum S58^T)^* (Hara et al., 2019).

Metagenomics of the kodo millet rhizosphere revealed that *Actinobacteria* were the most abundant component of the rhizobiome, among which *Frankia* were particularly abundant. *Frankia* are known to induce N-fixing nodules on actinorhizal host plants. Homocitrate synthase, nitrogenase, NifA, and VnfA were also identified, indicating the potential role of these communities in supporting plant N requirements under low-N conditions (Prabha et al., 2019). Diazotrophs with root-associative characteristics from the genera *Azospirillum*, *Azotobacter,* and *Klebsiella* that were isolated from the rhizosphere of pearl millet growing in nutrient-poor soil in a semi-arid region could significantly increase the total root and shoot N content under pot-culture conditions (Tiwari et al., 2003). Interestingly, a foxtail millet genotype–microbiota interaction network was found to contribute to phenotypic plasticity, and the microbially mediated growth effects on foxtail millet were dependent on host genotype (Wang et al., 2022a; 2022b).

### First-generation self-fertilizing cereal crops: Increasing the association of crops with N-fixing bacteria

**Figure 2:**
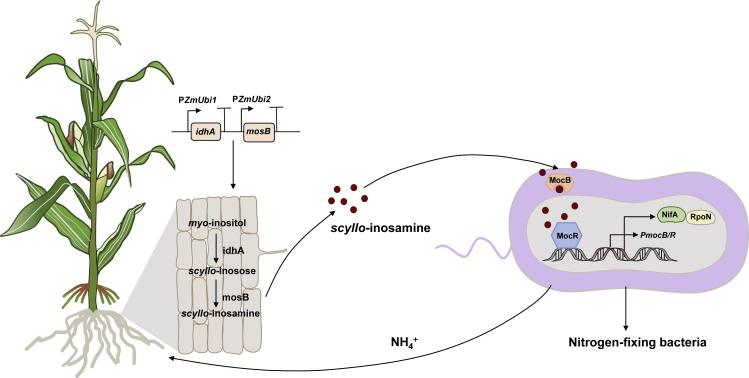
Engineering of associative nitrogen fixation between cereal plants and nitrogen-fixing bacteria. Engineered cereal plants (such as maize) have the ability to convert *myo*-inositol into *scyllo*-inosamine (rhizopine) via inositol dehydrogenase (IdhA) and aminotransferase (MosB). The nitrogen-fixing bacteria contain biosensor plasmids encoding the rhizopine-binding protein MocB and the rhizopine-dependent transcription factor MocR. These proteins drive the expression of genes placed downstream of the rhizopine-inducible promoter *PmocB*, such as genes encoding the nitrogenase NifA and nitrogen metabolism σ-factor RpoN, which enable biological nitrogen fixation after the rhizopine signal is perceived.

N-self-fertilizing cereal crops produce their own N nutrients or obtain them from their interaction partners. N-fixing organisms living freely in the rhizosphere or endophytically in plants provide large amounts of the combined N needed for cereal crops. However, the amount of fixed N from associative bacteria is not sufficient to completely support the growth and development of cereal crops. Therefore, we propose that the first generation of N-self-fertilizing crops should be created by improving associative interactions between N-fixing bacteria and cereal crops using synthetic biology techniques. One example is a synthetic barley that secretes rhizopines, secondary small molecular natural products, as described below (Figure 2) (Haskett et al., 2022).

#### Engineered barley plants secrete the rhizopine *scyllo*-inosamine

Rhizopines are symbiosis-specific compounds, including *scyllo-inosamine (SIA) and 3-O-methyl-scyllo-inosamine, that are* synthesized by a few rhizobia such as *S. meliloti* L5-30 (Bishop and Joerger, 1990) and serve as a carbon and N source for rhizobia. Rhizopine biosynthesis is regulated by the N-fixation master regulator NifA (Murphy et al., 1995). Rhizopines are excreted from the nodule and enhance the nodulation competitiveness of strains that carry the catabolic *moc* genes (Gordon et al., 1996). The *mosABC* genes are involved in rhizopine biosynthesis, and the *mocCABRDEF* genes participate in rhizopine catabolism in *S. meliloti* strain L5-30 (Murphy et al., 1987). Geddes et al. (2019) established a synthetic pathway for SIA in *Medicago truncatula* and barley by expressing *idhA* (encoding an inositol hydrogenase) and *mosB* (encoding a transaminase), thus producing SIA in engineered plants for the first time (Figure 2). This small molecule was sensed by a *Rhizobium* reporter strain, which induced the expression of the *moc* promoter, driving the expression of *gfp* or *luciferase* genes (Geddes et al., 2019).

#### N fixation of engineered rhizobia induced by engineered barley plants

Based on the above concept, a homozygous rhizopine-producing barley line and a mixed rhizobium uptake system were developed (Haskett et al., 2022). This system enhanced SIA sensing sensitivity 1000-fold in the model rhizobium *A. caulinodans* ORS571. Using this improved genetic circuit, a nitrogenase gene expression system was generated in which rhizopine-dependent NifA and RpoN (N metabolism σ-factor) drove nitrogenase gene expression *in vitro*, and *in situ* colonization of engineered barley roots by *A. caulinodans* ORS571 was successfully established (Figure 2). Although *in situ* nitrogenase activity was suboptimal compared with wild-type rhizobia, nitrogenase activation was observed only in bacteria associated with engineered barley roots. This study suggests that associative N fixation by endophytic or free-living bacteria could be enhanced to meet the N requirements of cereal crops using synthetic biology to modify bacterium–plant interactions. The use of first-generation N-self-fertilizing cereal crops is expected to reduce the need for N fertilizer application by 20%–40%.

### Second-generation self-fertilizing cereal crops: Transferring symbiotic N fixation into cereal plants

**Figure 3:**
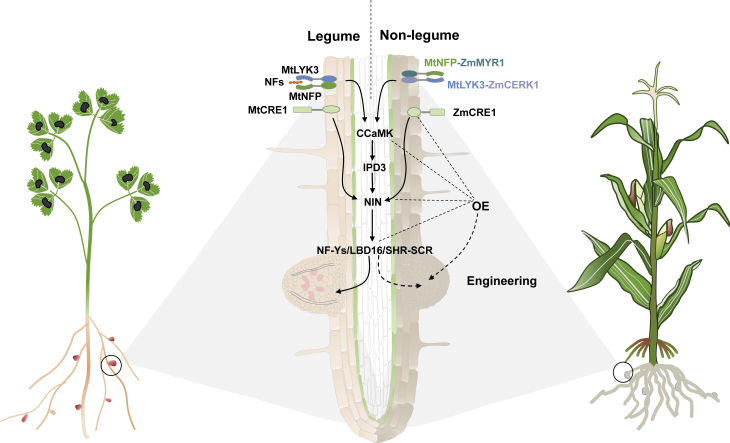
Engineering key regulators of nodule organogenesis in cereal crops to form nodule-like structures. In legume plants (such as *M. truncatula*, left) in low-nitrogen environments, roots release flavonoids to the rhizosphere to induce rhizobial secretion of NFs. NFs are recognized by the NF receptor complex comprising MtLYK3 and MtNFP on the plasma membrane, which activates the common symbiotic signaling pathway. The NF-induced calcium signal is decoded by CCaMK, which phosphorylates IPD3, ultimately inducing the expression of *NIN*/*NF-YS*/*LBD16*/*SHR-SCR* to initiate nodule formation. Non-legume crops (such as maize, right) are engineered for symbiotic nitrogen fixation by replacing the extracellular domains of the Myc factor receptors ZmMYR1 and ZmCERK1 with those of NF receptors MtNFP and MtLYK3, respectively. The chimeric receptors MtNFP–ZmMYR1 and MtLYK3–ZmCERK1 can perceive the NF signal and produce nodule-like structures by activating the common symbiotic signaling pathway and downstream components. Overexpression of genes encoding the cytokinin receptor ZmCRE1, CCaMK, and downstream transcription factors such as NIN, NF-Ys, LBD16, and the SHR–SCR module could also promote the division of cortical cells to form root-nodule-like structures. Dashed lines represent the potential mechanism for engineering cereal crops to form nodule-like structures.

Symbiotic N fixation is more efficient and makes a greater contribution to agricultural production than free-living and associative N fixation. However, symbiotic N-fixing systems require that diazotrophs specifically infect host plants to induce the formation of N-fixing organs, a process mediated by a highly complex signaling network between the symbiotic partners. The molecular mechanisms that control successful symbiosis between legumes and rhizobia have been revealed (Roy et al., 2020; Yang et al., 2022; Wang et al., 2022a; 2022b; Figure 3). Legume roots commonly release specific flavonoids into the soil, which attract rhizobia to the rhizosphere and stimulate them to produce and secrete NFs under low-N conditions. The NFs are then perceived by a plasma-membrane-localized receptor complex consisting of Nod Factor Perception (MtNFP) and LysM domain receptor-like kinase 3 (MtLYK3) in *Medicago truncatula* and Nod factor Receptor 1 (LjNFR1) and LjNFR5 in *Lotus japonicus*. Following activation of the common symbiosis signaling pathway, which is essential for the establishment of both root nodule symbiosis and arbuscular mycorrhizal symbiosis, the NF signal is conveyed to the nucleus to induce nucleus-associated calcium oscillations, thereby initiating downstream transcriptional responses. In the nucleus, the calcium signals are decoded by the chimeric Ca^2+^/calmodulin-dependent protein kinase CCaMK (MtDMI3/LjCCaMK). Activated MtDMI3/LjCCaMK then phosphorylates the transcriptional activator INTERACTING PROTEIN OF DMI3 (MtIPD3)/LjCYCLOPS. Phosphorylated IPD3 interacts with DELLAs (proteins with highly conserved DELLA amino acid motifs in their N-terminal domains) and NODULATION SIGNALING PATHWAY 1/2 to activate the expression of core transcriptional regulators, leading to root nodule symbiosis or arbuscular mycorrhizal symbiosis. NODULE INCEPTION (NIN), the core regulator of nodule organogenesis, plays a central role in integrating nodulation signaling and phytohormone (cytokinin and auxin) signaling during nodule organogenesis.

Cereal crops undergo arbuscular mycorrhizal (AM) symbiosis, which shares the common symbiosis signaling pathway with nodule symbiosis, providing the possibility that specific genes from the nodule symbiosis of legumes could be introduced into non-legume cereal crops by synthetic biology (Mus et al., 2016). We propose that such plants will represent the second generation of N-self-fertilizing cereal crops, which are expected to reduce the use of N fertilizer by ∼40%–90%.

#### Expression of NF receptors in rice

The LysM receptor heterodimer comprising OsMYR1 and OsCERK1 mediates the perception of signals from AM fungi in rice (He et al., 2019). A chimeric receptor pair was created in which the extracellular domains of OsMYR1 and OsCERK1 were replaced by those of the NF receptors MtNFP and MtLYK3. Interestingly, calcium spiking in response to NFs was observed when these chimeric constructs were expressed in rice (He et al., 2019). OsCERK1 and OsCEBiP are the major chitin elicitor-binding proteins in rice. Conversely, when the ectodomains of the NF receptors LjNFR1 and LjNFR5 were replaced by those of OsCERK1 and OsCEBiP in *L. japonicus*, symbiotic signaling was activated by chitin treatment in *nfr1-1*/*nfr5-2* mutants (Wang et al., 2014). Therefore, it is possible to engineer Myc factor receptors (Myc factors include lipochitooligosaccharides and short-chain chitooligosaccharides [CO4/CO5] secreted by AM fungi) in cereal crops for the perception and transduction of rhizobial NFs (Figure 3).

#### Expression of key regulators of nodule organogenesis in rice

NIN, a transcription factor induced by CYTOKININ RESPONSE 1-mediated cytokinin signaling in inner root cortical cells, is the transcriptional hub that integrates nodulation signaling and phytohormone signaling (especially cytokinin and auxin) for nodule organogenesis (Yang et al., 2022). NIN activates the expression of the Nuclear Factor-Y (NF-Y) subunit genes *NF-YA1* and *NF-YA2* (Soyano et al., 2013), which redundantly regulate the early stage of rhizobial infection and nodule meristem activity via transcriptional activation of *MtERN1* (Laloum et al., 2014). Overexpression of NIN and NF-Y subunit genes induced abnormal cortical cell division and spontaneous formation of root-nodule-like structures with an ontogeny similar to that of rhizobia-induced root nodules in the absence of rhizobial infection (Soyano et al., 2013). Gain-of-function mutations in MtDMI3/LjCCaMK and CYTOKININ RESPONSE 1 were also sufficient to trigger spontaneous root nodule organogenesis (Tirichine et al., 2006, 2007). Spontaneous nodulation was also observed when the kinase domain of CCaMK was expressed (Gleason et al., 2006; Shimoda et al., 2012; Takeda et al., 2012).

The developmental program of the root is recruited for root nodule organogenesis in legumes (Soyano et al., 2019, 2021). Auxin-responsive *LATERAL ORGAN BOUNDARIES*–*DOMAIN PROTEIN 16* (*LBD16*) is expressed in the root and promotes lateral root morphogenesis (Goh et al., 2012). An *LBD16*-mediated developmental program is co-opted downstream of NIN for nodule organogenesis (Schiessl et al., 2019; Soyano et al., 2019). Co-overexpression of *LBD16*/*NF-YA1*/*NF-YB1* induced the spontaneous formation of nodule-like structures in both the wild type and *nin* mutants (Soyano et al., 2019). The GRAS transcription factors SHORT ROOT (SHR) and SCARECROW (SCR) are key regulators of root development that control stem cell definition/maintenance and are expressed in the plant stem cell region and endodermis (Di Laurenzio et al., 1996; Helariutta et al., 2000). The MtSHR–MtSCR module is present in cortical cells of nodulating legumes, conferring on these cells the ability to de-differentiate and turning fully differentiated cortical cells into nodule primordia in response to symbiotic signals (Dong et al., 2021). Cortical cell division in response to cytokinin and NIN also requires the MtSHR–MtSCR module, and overexpression of *MtSHR*–*MtSCR* in both *M. truncatula* and rice led to the formation of root-nodule-like structures by inducing root cortical cell division. Thus, it is possible to generate nodule-like structures to accommodate N-fixing rhizobia by modulating the expression of these key regulators of nodule organogenesis in cereal crops (Figure 3). However, how microaerobic conditions are maintained to allow the rhizobia to perform N fixation in nodules remains to be elucidated.

### Third-generation N-self-fertilizing cereal crops: Engineering autonomous N fixation in cereal crops

The N-fixing gene cluster of *Paenibacillus* WLY78 consists of nine genes, including *nifB*, *nifH*, *nifD*, *nifK*, *nifE*, *nifN*, *nifX*, *hesA,* and *nifV*, and represents the smallest N-fixation operon identified to date. When this gene cluster was transferred into *Escherichia coli*, some nitrogenase activity was detected (Wang et al., 2013). In 2016, 28 genes from *Paenibacillus* WLY78 and *Klebsiella oxytoca* were cloned into two plasmids under the control of the *Paenibacillus nif* promoter. Expression of the *Paenibacillus suf* operon (Fe–S cluster assembly) and the potential electron transport genes *pfoAB*, *fldA*, and *fer* increased nitrogenase activity in *E. coli* (Li et al., 2016). Interestingly, an *E. coli* strain with 10 genes of the Mo-free alternative nitrogenase system of *Azotobacter vinelandii* was able to fix significant amounts of N (Yang et al., 2014). Expression of *nifSU* (Fe–S cluster assembly) and *nifFJ* (nitrogenase-specific electron transport) from *K. oxytoca* also enhanced nitrogenase activity. The mixed assembly of potential electron transport genes from *Paenibacillus* (*pfoA/B*, *fldA*) and *K. oxytoca nifSU* restored nitrogenase activity to 50.1% that of the parent strain (Li et al., 2016). These findings suggest that proteins that assist in Fe–S cluster assembly and electron transport are essential for nitrogenase activity. In addition, this study identified the smallest N-fixing gene cluster in a prokaryotic microorganism, with potential applications for the engineering of N-fixing genes in cereal crops.

#### Expression of nitrogenase subunits in mitochondria and chloroplasts

**Figure 4:**
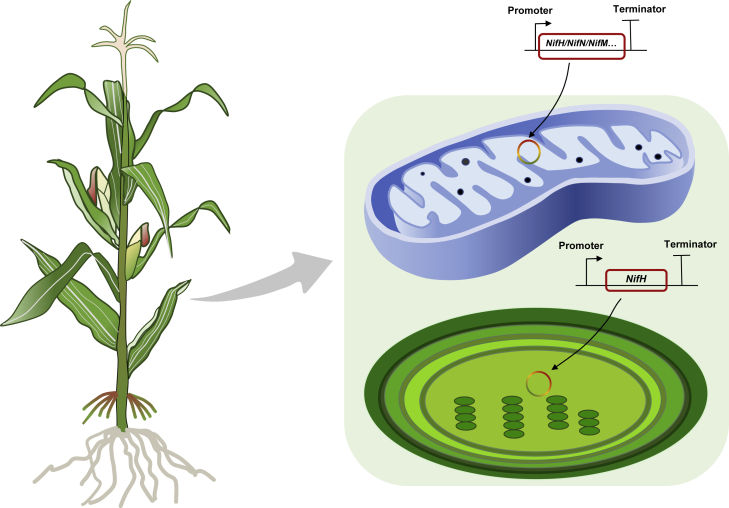
Engineering nitrogenase genes in the mitochondria and chloroplasts of cereal crops. The mitochondria and chloroplasts of cereal crops could provide a hypoxic environment for nitrogen fixation by introducing *nif* genes into these two organelles. A major challenge, in addition to determining whether the actual oxygen gas content in these organelles is at a level that maintains nitrogenase activity, is to determine how to introduce and successfully express optimized nitrogenase gene clusters in the organelles of cereal crops.

Because nitrogenase is extremely oxygen sensitive, some researchers have suggested that mitochondria and plastids might be suitable compartments for protecting nitrogenase from oxygen (Soto et al., 2013; Oldroyd and Dixon, 2014; Buren et al., 2017) (Figure 4). The *nifH* gene was successfully introduced into tobacco chloroplasts, and protein expression was achieved (Oldroyd and Dixon, 2014; Buren et al., 2017). The dinitrogen synthase reductase NifH and maturation enzyme NifM were successfully expressed in the yeast mitochondrial matrix, whereas expression of NifH in the cytoplasm required co-expression of NifM, NifU, and NifS under anaerobic conditions (Lopez-Torrejon et al., 2016). Nine *nif* genes (*nifH*, *nifD*, *nifK*, *nifU*, *nifS*, *nifM*, *nifB*, *nifE*, and *nifN*) of *Azotobacter vinelandii* were also successfully expressed in yeast mitochondria, and NifDK tetramers formed (Burén et al., 2017). Yang et al. (2017) utilized the modular approach of synthetic biology to evaluate the electron donors from chloroplasts, root plastids, and mitochondria as Mo nitrogenases and Fe nitrogenases and determined that electron transfer components from plant organelles can be used to support nitrogenase activity. Allen et al. (2017) also transferred the *nif* operon (16 genes) from *Klebsiella pneumoniae* into tobacco mitochondria, but the expressed proteins were inactive. Recently, the nitrogenase co-factor maturase NifB was successfully expressed in tobacco mitochondria/chloroplasts and rice mitochondria (He et al., 2022; Jiang et al., 2022).

#### Expression of the nitrogenase complex in the plant cytoplasm

Cheng et al. (2005) successfully expressed the *nif* operon in the green alga *Chlamydomonas reinhardtii*, but no nitrogenase activity was detected. In an exciting study, the minimal N-fixation gene cluster of *Paenibacillus polymyxa* was successfully transferred into the model plant *Arabidopsis thaliana.* The transgenic *Arabidopsis* expressing a nine-*nif* gene cassette (*nifBHDKENXhesAnifV*) showed moderate nitrogenase activity, resulting in higher biomass and chlorophyll contents compared with control plants grown in low-N or N-free medium, according to a manuscript available on the Research Square reprint server (Yao et al., 2021). The transgenic *Arabidopsis* also showed resistance to two nitrogenase substrates, KCN and NaN_3_. Overexpression of genes encoding electron transfer complex components resulted in higher N-fixation efficiency, consistent with the results of combined expression of *nif* genes in bacteria (Li et al., 2016). If the results of this study can be validated, this will provide the possibility to construct cereal crops capable of autonomous N fixation. We propose that such plants would represent the third generation of N-self-fertilizing cereal crops.

### Perspectives

Engineering N-fixing symbiosis between cereals and diazotrophic bacteria represents a promising strategy for the sustainable delivery of biologically fixed N to host cereal crops to enhance agricultural production. Although exciting progress has been made, many big challenges must be overcome before the engineering of BNF in non-legume plants becomes a reality.

#### Cultivation of new cereal varieties with symbiotic and autonomous N fixation

With rapid advances in our understanding of symbiotic N fixation mechanisms, plans to cultivate new, self-fertilizing cereal crop varieties have been put into practice in several laboratories worldwide. To achieve this goal, the following issues must be addressed. (1) How many symbiotic-nodulation-related genes should be transferred to the genomes of cereal crops? Although key regulatory genes that control root nodule development have been identified, genes associated with rhizobial infection and plant immune responses, as well as their underlying mechanisms, must be studied further. (2) Which tissues and organs of cereal crops are suitable for the expression of genes from legumes or other symbiotic N-fixing plants, and how can these gene expression levels be controlled? (3) How can microaerobic conditions be created to enable rhizobia to perform N fixation in the nodule organs of engineered cereal crops?

Synthetic biology provides another approach for introducing BNF into cereal crops to cultivate new varieties that can fix N autonomously. The recent successful expression of the minimal N-fixing gene cluster from *Paenibacillus* in *Arabidopsis* (Yao et al., 2021) represents a major breakthrough in N-fixation research. However, several issues remain to be clarified. (1) Constitutive expression of multiple exogenous genes with the same promoter often leads to gene silencing; solving this problem remains a major challenge. No other laboratory has yet reported the successful expression of the N-fixing minimal gene cluster in eukaryotic cells; is this due to gene silencing or other unknown factors? (2) Assuming that the exogenous minimal N-fixing gene cluster is successfully transferred to cereal crops, nitrogenase activity may be detected under N-deficient and microaerobic conditions, but the whole plant cannot always grow under hypoxic conditions. Therefore, how can the expression of the N-fixing gene cluster be induced in specific plant tissues and organs? (3) How can localized oxygen concentrations be regulated in transgenic plant tissues or organs to overcome the oxygen sensitivity of nitrogenase? Leghemoglobin and its regulators play a key role in protecting the nitrogenase complex from oxygen in the nitrogen-fixing root nodules of legumes (Larrainzar et al., 2020; Jiang et al., 2021). Can leghemoglobin be engineered in cereal crops to increase cytoplasmic nitrogenase activity in the future?

#### Manipulating associative diazotrophs for cereal crops

Cereal crops obtain 1/4 to 1/3 of their combined N supply from free-living and associative diazotrophs in some farmlands (Pankievicz et al., 2019; Soumare et al., 2020). Microbiomics has revealed that abundant rhizosphere and endophytic diazotrophs colonize major cereal crops such as maize and rice (Yoneyama et al., 2019). The principles of synthetic biology could be applied to modify N-fixing bacteria in the microbiomes of cereal crop rhizospheres to improve the N-fixing efficiency of the microorganisms, perhaps reducing the need for chemical N fertilizer application by half. A study was performed to transfer 12 *nif* gene clusters between 15 different bacterial species, including *E. coli* and 12 *Rhizobium* species, in an effort to design a bacterium for the delivery of high-flux N to cereal crops. In this study, cereal plants were engineered to produce multiple carbon sources (such as opine), and the corresponding catabolic pathways were transferred into microbes to generate synthetic symbiotic relationships (Ryu et al., 2020). Transgenic barley could synthesize a rhizopine to specifically induce the expression of nitrogenase genes in free-living or associative N-fixing bacteria, thereby affecting growth and development of the cereal crop (Haskett et al., 2022). CRISPR gene editing was recently used to create loss-of-function mutations of an apigenin catabolism gene in rice, which increased the biosynthesis and secretion of flavonoid molecules such as apigenin in rice roots, stimulated the formation of soil N-fixing bacterial biofilms, and altered the structure of the rice rhizosphere microbiome by recruiting N-fixing bacteria (Yan et al., 2022). The engineered rice plants increased soil N content and showed improved yields (Yan et al., 2022), demonstrating that BNF in soil can be changed by manipulating the flavonoid biosynthesis pathways of cereal crops. The combined application of microbiome and synthetic biology techniques could be used to help fulfill most of the combined N requirements of cereal crops.

## Funding

This work was supported by the 10.13039/501100001809National Natural Science Foundation of China (32070270, 32050081, 32088102, and 31825003) and the Chinese Academy of Sciences Project for Young Scientists in Basic Research (YSBR-011).
